# SIK2 promotes ovarian cancer cell motility and metastasis by phosphorylating MYLK

**DOI:** 10.1002/1878-0261.13208

**Published:** 2022-03-25

**Authors:** Xiu Shi, Xuejiao Yu, Juan Wang, Shimin Bian, Qiutong Li, Fengqing Fu, Xinwei Zou, Lin Zhang, Robert C. Bast, Zhen Lu, Lingchuan Guo, Youguo Chen, Jinhua Zhou

**Affiliations:** ^1^ Department of Obstetrics and Gynecology The First Affiliated Hospital of Soochow University Suzhou China; ^2^ 12582 Clinical Research Center of Obstetrics and Gynecology Jiangsu Key Laboratory of Clinical Immunology Soochow University Suzhou China; ^3^ Jiangsu Institute of Clinical Immunology The First Affiliated Hospital of Soochow University Suzhou China; ^4^ Department of Imaging Department The First Affiliated Hospital of Soochow University Suzhou China; ^5^ Department of Experimental Therapeutics University of Texas M.D. Anderson Cancer Center Houston TX USA; ^6^ Department of Pathology The First Affiliated Hospital of Soochow University Suzhou China

**Keywords:** cell motility, metastasis, MYLK, ovarian cancer, phosphorylation, SIK2

## Abstract

Salt‐inducible kinase 2 (SIK2; also known as serine/threonine‐protein kinase SIK2) is overexpressed in several cancers and has been implicated in cancer progression. However, the mechanisms by which SIK2 regulates cancer cell motility, migration and metastasis in ovarian cancer have not been fully discovered. Here, we identify that SIK2 promotes ovarian cancer cell motility, migration and metastasis *in vitro* and *in vivo*. Mechanistically, SIK2 regulated cancer cell motility and migration by myosin light chain kinase, smooth muscle (MYLK)‐meditated phosphorylation of myosin light chain 2 (MYL2). SIK2 directly phosphorylated MYLK at Ser343 and activated its downstream effector MYL2, promoting ovarian cancer cell motility and metastasis. In addition, we found that adipocytes induced SIK2 phosphorylation at Ser358 and MYLK phosphorylation at Ser343, enhancing ovarian cancer cell motility. Moreover, SIK2 protein expression was positively correlated with the expression of MYLK‐pS343 in ovarian cancer cell lines and tissues. The co‐expression of SIK2 and MYLK‐pS343 was associated with reduced median overall survival in human ovarian cancer samples. Taken together, SIK2 positively regulates ovarian cancer motility, migration and metastasis, suggesting that SIK2 is a potential candidate for ovarian cancer treatment.

## Graphical abstract

SIK2 is overexpressed in several cancers and has been implicated in cancer progression. However, the mechanisms by which SIK2 regulates cancer cell motility and metastasis in ovarian cancer (OC) have not been fully described. Here, we have identified that SIK2 promotes OC cell motility and metastasis by phosphorylating MYLK/MYL2, suggesting that SIK2 may serve as a potential target in OC treatment.

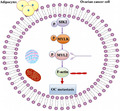

**Abbreviations**
ADP: adenosine diphosphate
ATP: adenosine triphosphate
BSA: bovine serum albumin
Co‐IP: co‐immunoprecipitation
EV: empty vector
F‐actin: filamentous actin
FBS: fetal bovine serum
GAPDH: glyceraldehyde 3‐phosphate dehydrogenase
IHC: immunohistochemistry
MS: mass spectrometry
MYL2: myosin light chain 2
MYLK: myosin light chain kinase
MYPT: myosin phosphatase‐targeting protein
NMII: non‐muscle myosin II
OD: optical density
OE: overexpression
PBS: phosphate‐buffered saline
PCR: polymerase chain reaction
ROCK: rho‐associated coiled‐coil‐containing kinase
SIK2: salt‐inducible kinase 2
siRNA: small interfering RNA
WB: western blot
WT: wild‐type

## 1 Introduction

Ovarian cancer is one of the most common malignant tumours of the female reproductive system [1]. A majority of patients with ovarian cancer are diagnosed at an advanced stage, presenting with extensive metastasis within the pelvic and peritoneal cavities [2].

Metastasis of ovarian cancer occurs when cells from the primary tumour are shed and disseminated directly to the surface of the peritoneum and omentum, or to other parts of the body through the lymphatic system or the blood stream [3, 4]. There are differences in the mechanisms of cancer metastasis through peritoneal dissemination, lymphatic spread and hematogenous dissemination. However, all these processes are driven by increased cell motility, which involves cycles of actin polymerization, cell adhesion and actomyosin contraction [5].

Salt‐inducible kinase 2 (SIK2) is a serine/threonine protein kinase with a well‐established role in the regulation of metabolism [6, 7]. Findings from a recent study that we conducted showed that SIK2 is required for centrosome splitting in the mitosis of ovarian cancer cells [8] and that ARN‐3236 inhibited SIK2 and sensitized ovarian cancer cells to paclitaxel [9]. SIK2 is also required for adipocyte‐induced proliferation of metastatic ovarian cancer [10]. However, the exact role of SIK2 in cancer cell motility, migration and metastasis is not fully understood.

Non‐muscle myosin II (NMII) plays an important role in cell migration and adhesion by moving the cell body forward and retracting the rear of the cell, largely via the interaction with filamentous actin (F‐actin) [11]. NMII consists of two 230‐kDa heavy chains, two 20‐kDa regulatory light chains (RLCs, encoded by MYL2 gene) and two 17‐kDa essential light chains that maintain the heavy‐chain structure. The phosphorylation of MYL2 at Ser19 regulates NMII activity by inducing conformational changes of the myosin heads [11]. Several kinases have been reported to phosphorylate MYL2 of NMII, including Rho‐associated coiled‐coil kinase (ROCK), zipper‐interacting protein kinase (ZIPK), myotonic dystrophy kinase‐related CDC42‐binding kinase (MRCK) and myosin light chain kinase (MYLK) [12, 13, 14]. The specific mechanism has not been fully elucidated.

In this study, we demonstrated that SIK2 regulates cell motility, migration and metastasis by phosphorylating MYLK in ovarian cancer.

## 2 Materials and methods

### 2.1 Ethics statement

The protocols for handling paraffin‐embedded ovarian cancer specimens, as well as fresh omentum samples were approved by the Ethics Committee of The First Affiliated Hospital of Soochow University (Suzhou, Jiangsu Province, China). Written informed consent forms were signed by all the enrolled patients. A case number was used to register all tissue samples in the database with no link to patient names or personal information.

### 2.2 Cell culture

Human ovarian cancer cell lines SKOV3 and OVCAR3, and breast cancer cell lines Hs578t and MDA‐MB‐231 were purchased from the Tumor Cell Bank of the Chinese Academy of Medical Sciences (Shanghai, China). OVCAR5, OVCAR8 and OVISE were provided by Tongji Medical College, Huazhong University of Science and Technology. All cell lines were confirmed by short tandem repeat DNA fingerprinting. The cells were cultured in a humidified incubator with 5% (v/v) CO_2_ at 37 °C. Hs578t and MDA‐MB‐231 were cultured in high‐glucose Dulbecco's modified Eagle's medium (HyClone Laboratories, Logan, UT, USA). Other cells were cultured in RPMI‐1640 medium (HyClone Laboratories) with 10% fetal bovine serum (FBS; Biosera, Shanghai, China) and 1% penicillin/streptomycin (Beyotime Institute of Biotechnology, Shanghai, China).

### 2.3 Transfection

Cells were transfected with control short interfering RNA (siRNA) using Lipofectamine™ 3000 reagent, according to the manufacturer’s protocol (Invitrogen, Carlsbad, CA, USA). A mixture of siRNA (25 nm final concentration) and Lipofectamine™ 3000 reagent was incubated for 20 min at room temperate before being applied to the cells. Plasmids transfection (5 µg of DNA each) was performed using Lipofectamine™ LTX Reagent with PLUS™ Reagent, following the manufacturer’s instruction (Invitrogen, Carlsbad, CA, USA).

### 2.4 Western blotting

Proteins were extracted from cultured cells with radio‐immunoprecipitation assay lysis buffer (Beyotime Institute of Biotechnology), and concentration was determined using the BCA Protein Assay Kit (Beyotime Institute of Biotechnology). An equal amount of total protein was boiled in the sample buffer and separated by sodium dodecyl sulfate–polyacrylamide gel electrophoresis (SDS/PAGE), and transferred onto a polyvinylidene difluoride (PVDF) membrane (Millipore, Bedford, MA, USA) using a wet transfer system (Bio‐Rad, Hercules, CA, USA). The membranes were incubated with the indicated antibodies. GAPDH was used as an endogenous control in each immunoblotting experiment.

### 2.5 Wound healing assay

Wound healing assay was employed to assess cell migration. At 48 h after transfection with siRNA, a pipette tip was used vertically to scratch a horizontal line in the middle of the six‐well plate. Afterwards, the cells were washed with PBS thrice to remove the scratched cells, and serum‐free medium was subsequently added. Wound healing ability of the cells were evaluated at 0 and 24 h using an inverted phase‐contrast microscope (IncuCyte^®^; Essen BioScience, Ann Arbor, MI, USA).

### 2.6 Transwell migration and invasion assays

Transwell migration assay was conducted in 24‐well transwell chambers (Corning Life Sciences, Corning, NY, USA) containing filters with 8‐μm pores without Matrigel. Cell invasion was evaluated using a transwell assay with Matrigel (Corning Life Sciences). Approximately 5 × 104 cells in 100 μL of serum‐free RPMI‐1640 medium were placed in the upper chamber, and 500 μL of the medium with 20% FBS was added to the lower chamber. Plates were incubated in 5% CO_2_ at 37 °C for 12 h for the transwell migration assay and 24 h for the invasion assay, and cells were subsequently fixed in paraformaldehyde for 20 min and stained with crystal violet (Beyotime Institute of Biotechnology) for 20 min. Cells on the upper side of filters were detached with cotton swabs, and the filters were washed with PBS thrice. Finally, cells on the lower side of the filters were counted under a microscope (Olympus IX81; Olympus, Tokyo, Japan) at 200× magnification.

### 2.7 Immunofluorescence and phalloidin staining of F‐actin

Actin‐stain™ 670 phalloidin (Cytoskeleton, Denver, CO, USA) was used for cytoskeletal F‐actin staining. Approximately 2 × 104 cells in 1 mL of RPMI‐1640 medium with 10% FBS were seeded on a 20‐mm confocal dish. On the next day, the cells were gently washed once with PBS at 37 °C and fixed in a fixative solution for 10 min. After washing, permeabilizing and blocking, 200 µL of 200 nm Actin‐stain™ 670 phalloidin was added to the cells and they were incubated in the dark at room temperature for 30 min. For SIK2 and p‐MYL2 staining, the primary antibody was incubated overnight at 4 °C, followed by the incubation of the fluorescent dye‐conjugated secondary antibody (Cell Signaling Technology, Danvers, MA, USA) for 1 h. Subsequently, DNA was counterstained for 30 s with 200 µL of 100 nm DAPI in PBS. Fluorescent images were visualized using a laser confocal microscope (LSM880; Carl Zeiss, Oberkochen, Germany).

### 2.8 Antibodies and reagents

Primary antibodies against SIK2, F‐actin, MYL2, phospho‐MYL2, ROCK1 and ROCK2 were purchased from Cell Signaling Technology. MYLK antibody, SIK2‐pS358 antibody and GAPDH antibody were purchased from Abcam (Cambridge, MA, USA), Kinexus (Vancouver, BC, Canada) and Sigma‐Aldrich (St. Louis, MO, USA), respectively. SIK2 inhibitor, ARN‐3236, was purchased from Selleckchem (Houston, TX, USA), while MYLK inhibitor, ML‐9, was purchased from Santa Cruz Biotechnology (Dallas, TX, USA). Human gateway entry clone empty vector and MYLK (Human Gene and Protein Database clone number: MYLK) were obtained from Shanghai Integrated Biotech Solutions Company (Shanghai, China).

### 2.9 *In vitro* kinase assay

SIK2 kinase reactions were assessed using a reaction mixture that contained SIK2 recombinant protein (15 nm), ATP (10 µm), and different concentrations of the peptide or protein substrate. The SIK2 assay was performed as previously described [9]. The results were plotted and analyzed using the graphpad prism 7 software (GraphPad Software, Petaluma, CA, USA).

### 2.10 Nanoflow liquid chromatography–tandem mass spectrometry (LC–MS/MS)

Recombinant SIK2 protein (Abcam, Cambridge, UK) and wild‐type MYLK peptide (aa330‐355) or MYLK mutant peptide (S343A) (synthesized by Shanghai Apeptide, Shanghai, China) were mixed together with ATP (Cell Signaling Technology) and kinase buffer (Cell Signaling Technology) in 1.5‐mL eppendorf tubes. After incubation at room temperature for 30 min, the two samples were analyzed on an EASY‐nLC™ 1000 coupled to a LTQ Orbitrap Elite mass spectrometer (Thermo Fisher Scientific, Grand Island, NY, USA) equipped with a nanoelectrospray source. Peptides were separated on a 15‐cm analytical column (Acclaim PepMap 100 C18 RSLC column, 2 µm, 150 × 0.050 mm, Thermo Fisher Scientific). Each sample was autosampled and separated using a 60‐min gradient at a flow rate of 300 nL·min^−1^ from 0% buffer B (99.99% ACN and 0.1% formic acid) to 5% B in 1 min, followed by a linear gradient from 5% B to 25% B in 45 min, ramping to 40% B in 5 min, increasing up to 95% B within 2.5 min, and maintained for 6.5 min. The LTQ Orbitrap Elite mass spectrometer was operated in a data‐dependent acquisition mode. Spray voltage, S‐lens RF level and heated capillary temperature were set to 2 kV, 63.9% and 250 °C, respectively. Scans with an *m*/*z* range of 300–2000 were collected in positive polarity mode. Full scans were analyzed with 60 000 resolution at *m*/*z* = 400 and predicted AGC target of 10. Tandem mass spectra were extracted using Mascot Distiller 2.7 (Matrix Science Inc, London, UK). MS/MS samples were analyzed using Mascot version 2.5.1 (Matrix Science Inc). Mascot was set up to search the modified MYLK database (Q15746), assuming that no digestion enzyme existed, and the search was performed with a fragment ion mass tolerance of 0.050 Da and a parent ion tolerance of 10.0 PPM. Methionine oxidation and serine/threonine phosphorylation were specified as variable modifications.

### 2.11 Anti‐MYLK‐pS343 antibody preparation

MYLK‐pS343‐specfic antibody was prepared by Hua’an Biotechnology (Hangzhou, China). The MYLK‐pS343 peptide was first coupled to keyhole limpet hemocyanin and BSA, and then administered through multiple subcutaneous injections to immunize New Zealand white rabbits. Thereafter, blood was collected from the rabbits and the presence of phosphospecific antibodies in the serum was evaluated using enzyme‐linked immunosorbent assay (ELISA) and western blot. For final antibody purification, the serum was eluted through the phosphopeptide column and then passed through a non‐phosphopeptide column to obtain a phosphospecific antibody.

### 2.12 Dot blot assay

For the dot blot assay, peptides were diluted with TBS buffer and spotted onto nitrocellulose membranes, and the membranes were blocked with 5% skimmed milk diluted in tris‐HCl buffer solution tween buffer after drying at room temperature, followed closely by probing with the indicated antibodies, as previously described for immunoblotting analysis.

### 2.13 Co‐immunoprecipitation

Cells were lysed in 0.5 mL of lysis buffer, followed by at 14 000 ***g*** for 10 min at 4 °C. Lysates were then incubated with 5 μg of anti‐SIK2 antibody (Cell Signaling Technology) for 4 h, followed by centrifugation at 4 °C in the presence of 5 μL of prewashed protein‐A/G beads (Absin Bioscience, Shanghai, China). The beads were collected using a magnet and washed thrice with 1 mL of wash buffer. The immune‐precipitates were analyzed using SDS/PAGE and the proteins were captured using anti‐SIK2 (Cell Signaling Technology) and anti‐MYLK antibodies (Santa Cruz, Carlsbad, CA, USA).

### 2.14 Isolation of adipocytes

Fresh omental tissues were obtained from surgically resected normal and metastasis‐free omentum of patients with early‐stage malignant epithelial ovarian tumour. The study methodologies conformed to the standards set by the Declaration of Helsinki. The omental tissues were examined under a microscope by a pathologist to exclude metastasis. The isolation of adipocytes was performed as previously described [10]. The adipocytes present in the remaining upper yellow layer were co‐cultured with ovarian cancer cells.

### 2.15 Tissue microarray and immunohistochemistry

Formalin‐fixed, paraffin‐embedded ovarian cancer tissue microarray chips containing 144 tissue samples, together with their clinicopathological and prognostic information, were purchased from Shanghai Outdo Biotechnology (Shanghai, China). All samples were clinically and pathologically judged to be correctly tagged. The tissue chips were dried at 60 °C for 1 h, deparaffinized, rinsed with pure water thrice and heat‐repaired for 20 min using EDTA repair solution. Subsequently, the tissue chips were blocked for 10 min and rinsed thrice with phosphate‐buffered saline (PBS). Additionally, the tissue chips were incubated with SIK2 antibody (1 : 200, ab53423; Abcam, Cambridge, UK) and p‐MYLK antibody (1 : 500; Hangzhou HuaAn Biotechnology) separately for 30 min at room temperature and were rinsed thrice with PBS for not < 1 min each time. The secondary antibody was incubated at room temperature for 30 min, developed with 3,3'‐diaminobenzidine as the colour‐developing agent for 5 min and subsequently rinsed with tap water for 5 min; then, Hastelloy^®^ hematoxylin counterstain (Sigma‐Aldrich) was added for 1 min. After that, it was immersed in 0.25% hydrochloric acid alcohol for no < 2 s, rinsed with tap water for more than 2 min and dried at room temperature before mounting.

### 2.16 Lentivirus infection

SIK2 knockdown lentivirus with green fluorescent protein and lentivirus with luciferase was purchased from Shanghai GeneChem Company. The sequence of shSIK2(in pGV493) was as follows: 5‐ GCAGTTGTTGTATGAACAAAT‐3. To develop stable SIK2 knockdown sublines, SKOV3 and OVCAR8 cells were infected with viral supernatant for 24 h. For overexpression, SIK2 overexpression plasmid vector (PLX302‐SIK2) and control empty vector (pLX302‐EV), which were kind gifts from Dr. Ahmed Ashour Ahmed (University of Oxford, UK), were utilized. The plasmids were inserted into pRLenti virus vector (Obio Technology, Shanghai, China). Cells were infected with SIK2 lentivirus for 48–72 h. Puromycin was added to the sieve for one week. Then, the transfection efficiency was observed under the fluorescent microscope (Olympus IX81; Olympus). According to the above experimental method, the successfully constructed SIK2 knockdown and overexpression stable transfected cells were transfected again with luciferase lentivirus.

### 2.17 *In vivo* orthotopic xenograft model

Female BALB/c‐Ighb severe combined immune‐deficiency (C.B‐17 SCID) mice (6–8 weeks old) were purchased from Shanghai Slac Laboratory Animal Co., Ltd and kept in the standard animal facility room. All animal experiments have been approved by the Animal Care and Use Committee of Soochow University (No. SCXK2017‐0006). Two orthotopic models of ovarian cancer metastasis (SKOV3 and OVCAR8 cancer cells) were chosen to evaluate the potential role of SIK2 in cancer cell motility and metastasis *in vivo*. OVCAR8‐shNC, OVCAR8‐shSIK2, SKOV3‐EV and SKOV3‐SIK2 OE cell subclones were orthotopically implanted in the right ovary bursa of C.B‐17 SCID mice. Sixteen C.B‐17 SCID mice were randomly divided into groups (eight mice/group) for OVCAR8 cancer cells. One group was injected with 2 × 10^6^ OVCAR8‐shNC cells expressing luciferase in the right ovary, while the second group was injected with 2 × 10^6^ OVCAR8‐shSIK2 cells in the right ovary. Ten C.B‐17 SCID mice were randomly divided into groups (five mice/group) for SKOV3 cancer cells. One group was injected with 5 × 10^6^ SKOV3‐EV cells expressing luciferase in the right ovary, while the other group was injected with 5 × 10^6^ SKOV3‐SIK2 OE cells in the right ovary. Four weeks after injection of the cells, the primary and metastatic tumours were evaluated using luciferase imaging. The tumour was observed using an In Vivo IVIS Spectrum Imaging System (PerkinElmer Ltd., Waltham, MA USA). After intraperitoneal injection of d‐luciferin (Thermo Scientific Pierce, Waltham, MA, USA) in PBS (15 mg·mL^−1^) at a dose of 10 µL·g^−1^ for 10 min, the mice were imaged for 5 min of exposure time. IVIS Living Image 4.2 was used to perform bioluminescence signals quantified as average radiance (photons·s^−1^·cm^−2^·sr^−1^). Mice were anaesthetized by the inhalation of 2% fluothane in oxygen/nitrous oxide. The tumours were harvested and the expression levels of SIK2, MYL2, MYL2‐pS19, MYLK and MYLK‐pS343 were compared between the two groups by western blotting and immunohistochemistry assays.

### 2.18 Statistical analysis

Experimental values were expressed as mean ± SD from three independent experiments. Statistical analyses were performed using spss 25 (SPSS Inc., Chicago, IL, USA). Statistical values were calculated by student *t*‐test (unpaired two‐tailed) for two groups or one‐way ANOVA for groups more than two. graphpad prism 7.0 was used for tests of multiple comparisons. Correlations between measured variables were analyzed using the Spearman rank correlation test. *P* < 0.05 was considered statistically significant: *P < 0.05, **P < 0.01, ***P < 0.001 and ****P < 0.0001.

## 3 Results

### 3.1 SIK2 promotes cell motility and metastasis in ovarian cancer

**Fig. 1:**
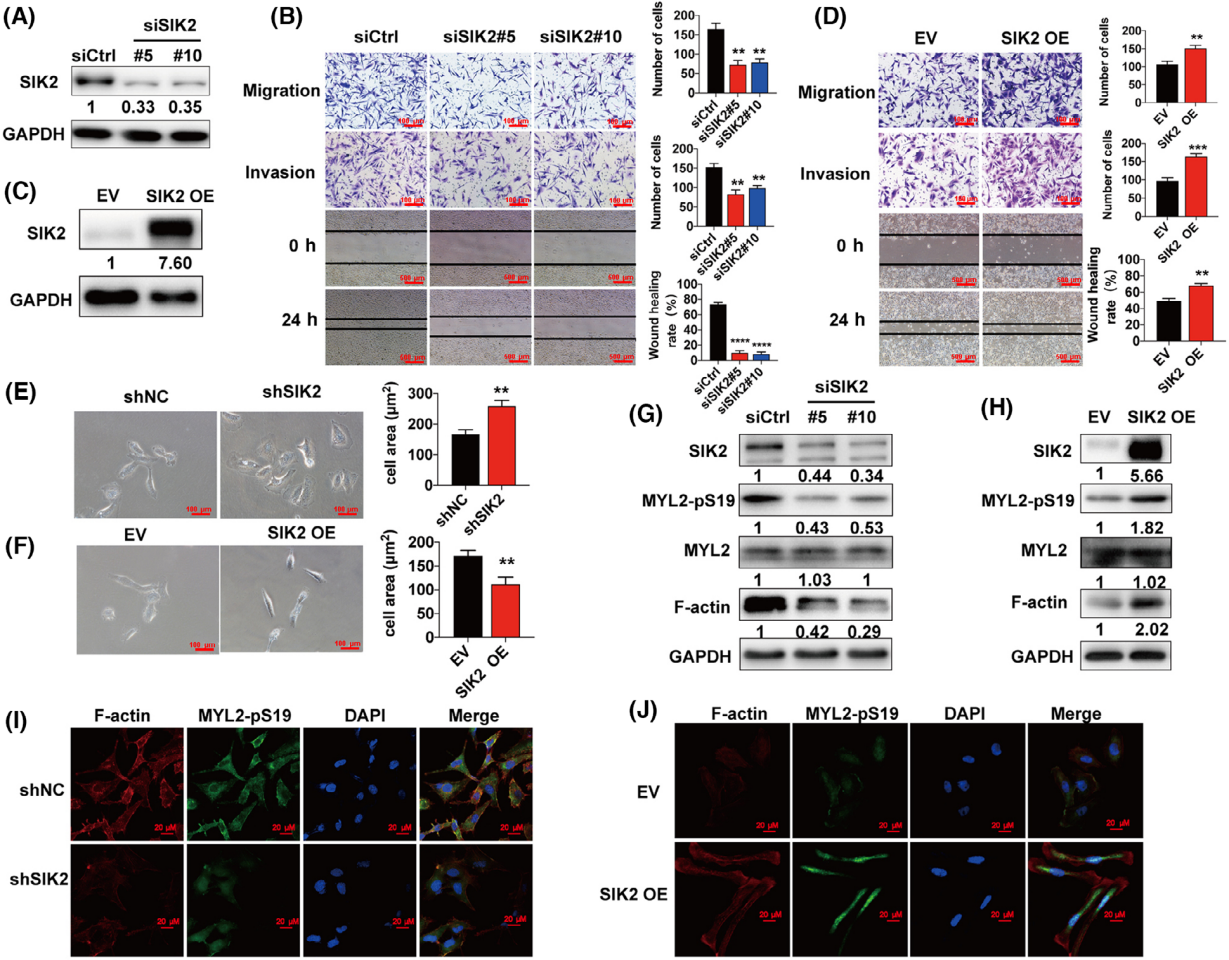
SIK2 promotes cell motility and metastasis in ovarian cancer. (A) SKOV3 cells were transfected with SIK2 siRNA for 48 h and knockdown efficiency was determined using western blot. (B) Wound healing as well as transwell migration and invasion assays were conducted in SKOV3 cells transfected with SIK2 siRNA; the representative images are shown on the left panel, while the quantitative values, which are presented as mean ± SD of three independent experiments, are shown on the right panel. Statistical values were calculated by one‐way ANOVA. (C) The expression levels of SIK2 were determined in SKOV3 cells transfected with empty vector (EV) or SIK2 overexpression virus (SIK2 OE) for 48 h using western blot. (D) Transwell and wound healing assays were performed using SKOV3‐SIK2 OE and SKOV3‐EV cell subclones. The representative images are shown on the left panel, while the quantitative values, which were presented as means ± SD of three independent experiments, are shown on the right panel. Statistical values were calculated by student *t*‐test (unpaired two‐tailed) for two groups. (E) imagej software (NIH, Bethesda, MD, USA) was used to measure the cell areas of SKOV3 cell subclones with stable expression of shSIK2 or shNC. The representative images are shown on the left panel, while the right panel shows the areas of 100 cells in each group presented as means ± SD. Statistical values were calculated by Student *t*‐test (unpaired two‐tailed). (F) The cell area of SKOV3–EV and SKOV3–SIK2 were measured using imagej. The representative images are shown on the left panel, while the right panel shows the areas of 100 cells in each group presented as means ± SD. Statistical values were calculated by Student *t*‐test (unpaired two‐tailed) for two groups. (G) Immunoblotting of F‐actin and MYL2‐pS19 in SKOV3 after transfection with SIK2 siRNA for 72 h. (H) Immunoblotting of F‐actin and MYL2‐pS19 in SKOV3–EV and SKOV3–SIK2. (I) SKOV3‐shSIK2 cells were stained for F‐actin (red), MYL2‐pS19 (green), and DAPI (blue), with the SKOV3‐shNC cells being used as the controls. (J) SKOV3–EV and SKOV3–SIK2 cell subclones were stained using anti‐F‐actin (red), anti‐MYL2‐pS19 (green), and DAPI (blue); scale bar = 20 μm for 40×. SIK2 OE represents SIK2 overexpression. All the experiments were repeated in three independent experiments. Bar plots represent the means ± SD (**P < 0.01, ***P < 0.001, ****P < 0.0001).

To determine if SIK2 affects cancer cell motility, siRNA transfection was used to deplete SIK2 in ovarian cancer cell lines (SKOV3 and OVCAR8) and breast cancer cell lines (MDA‐MB‐231 and Hs578t). The significant reduction in SIK2 protein expression was an indication that the siRNA‐induced depletion of SIK2 had been successful in SKOV3 (Fig. 1A), OVCAR8, MDA‐MB‐231 and Hs578t (Fig. S1A). The SIK2–depleted cells were then used to conduct wound healing and transwell assays. The results from these assays suggested that the capacity for migration and invasion by SKOV3 (Fig. 1B), OVCAR8, MDA‐MB‐231 and Hs578t (Fig. S1B) was significantly inhibited after the knockdown of SIK2. In contrast, SKOV3 cells with SIK2 overexpression showed substantially enhanced capacity for cell migration and invasion (Fig. 1C,D). To further investigate the effect of SIK2 on ovarian cancer motility, cells with stable SIK2 knockdown or SIK2 overexpression were engineered using lentivirus infection. Interestingly, we found that ovarian cancer cells with SIK2 knockdown had increased cell area (*P* = 0.0025 for SKOV3, Fig. 1E and *P* = 0.0015 for OVCAR8, Fig. S1C), while cells with SIK2 overexpression exhibited reduced cell area (*P* = 0.005 for SKOV3, Fig. 1F), indicating a contractile phenotype. These results indicate the possible role of SIK2 in determining cell contractility and motility.

The force required for the cell motility process is generated by the actin cytoskeleton [15]. Actin filaments (F‐actin) are the main components of the actin cytoskeleton. NMII, which interacts with F‐actin in non‐muscle cells, is regulated by the phosphorylation or dephosphorylation of its regulatory light chain, MYL2 [16]. Consequently, we investigated the effect of SIK2 on F‐actin and MYL2‐pS19. The results from western blot showed that there was a significant reduction in the expression of F‐actin and MYL2‐pS19 after siRNA‐induced knockdown of SIK2 (Fig. 1G and Fig. S1D), while SIK2 overexpression significantly increased the expression of F‐actin and MYL2‐pS19 (Fig. 1H). Moreover, SIK2 knockdown resulted in less intense cortical F‐actin and MYL2‐pS19 staining in SKOV3 and OVCAR8 cells by immunofluorescent staining (Fig. 1I and Fig. S1E,F). These results were consistent with those obtained using the breast cancer cell lines, MDA‐MB‐231 and Hs578t (Fig. S1F). In contrast, SIK2 overexpression in SKOV3 cells was associated with more intense cortical F‐actin and MYL2‐pS19 immunofluorescent staining (Fig. 1J). These results suggest that SIK2 regulates p‐MYL2‐mediated cell motility and contractility.

### 3.2 SIK2 regulates cell motility and MYL2 phosphorylation through MYLK

**Fig. 2:**
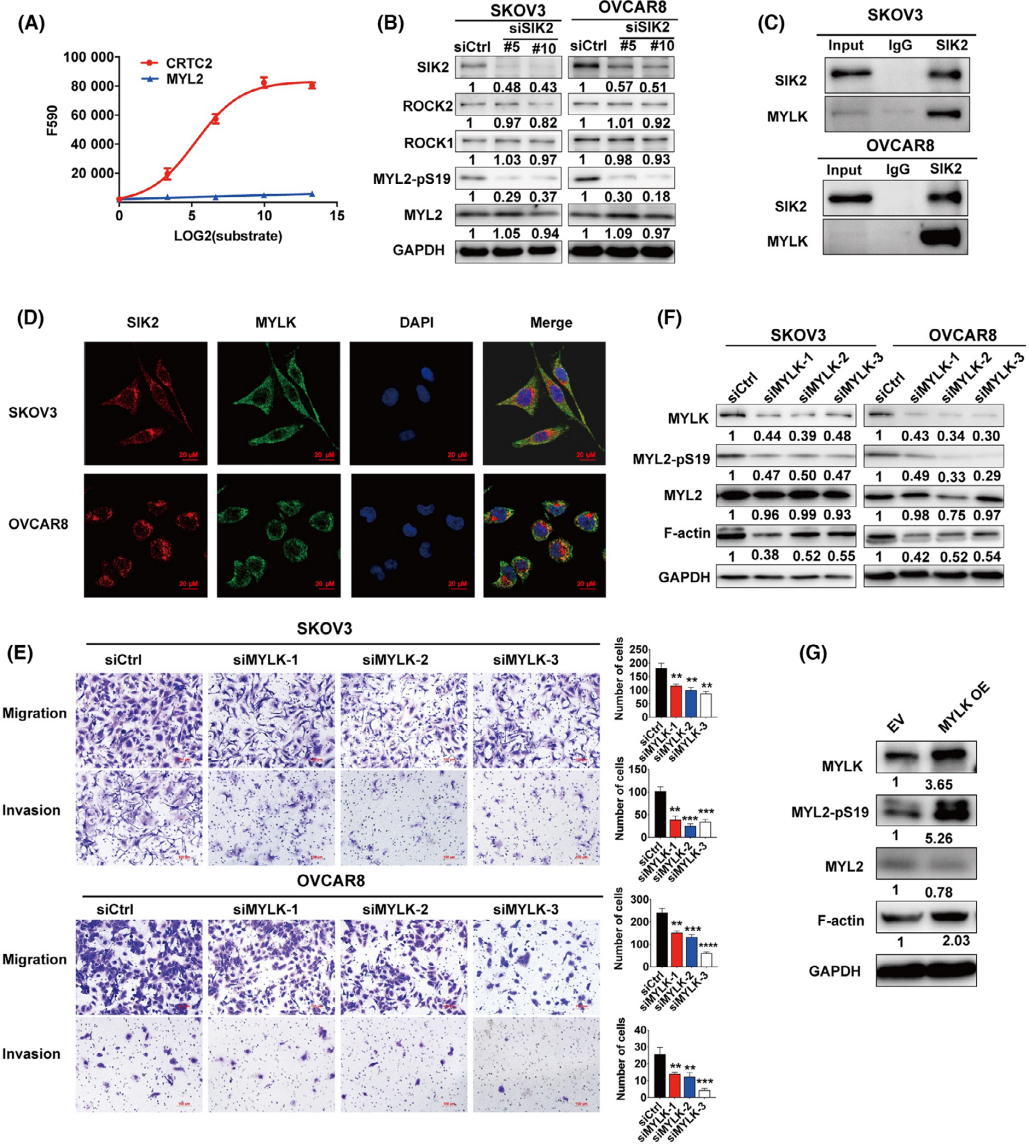
SIK2 regulates cell motility and MYL2 phosphorylation through MYLK. (A) *In vitro* kinase assay with recombinant SIK2 protein and CRTC2‐derived peptide (red) and MYL2 peptide (blue). (B) Immunoblotting assay of F‐actin, MYL2‐pS19, ROCK1 and ROCK2 after transfection with SIK2 siRNA for 72 h. (C) Cell lysates were first immunoprecipitated using anti‐SIK2 or IgG followed by detection of MYLK expression using western blot. (D) The representative images of immunofluorescent staining for SIK2 (red) and MYLK (green) in SKOV3 and OVCAR8 cells; scale bar = 20μm for 40x. (E) The cell invasion and migration ability of SKOV3 and OVCAR8 cells was determined after MYLK siRNA transfection for 48 h using transwell assays with or without Matrigel. Representative images are shown on the left panel, and the results of quantitative analysis were presented as mean ± SD of three independent experiments. Statistical values were calculated by one‐way ANOVA for groups more than two. (F) Immunoblot analysis of F‐actin and MYL2‐pS19 expression in SKOV3 and OVCAR8 at 72 h after MYLK siRNA transfection. (G) Immunoblot analysis of F‐actin and MYL2‐pS19 expression in the OVCAR8‐shSIK2 subclone after transfection with control empty vector plasmid (EV) and MYLK overexpressing plasmid (MYLK OE) for 72 h. All the experiments were repeated in three independent experiments. Bar plots represent the means ± SD (**P < 0.01, ***P < 0.001, ****P < 0.0001).

To investigate whether SIK2 directly phosphorylates MYL2 at Ser19, an *in vitro* kinase assay was carried out using recombinant SIK2 protein and MYL2‐derived peptide (aa 10–30). Since CREB regulated transcription coactivator 2 (CRTC2) is a direct substrate of SIK2 and is involved in metabolism [9], CRTC2‐derived peptide (aa 166–175) was used as a positive control in the kinase assay. The results showed that the recombinant SIK2 protein did not phosphorylate the MYL2‐derived peptide (Fig. 2A), indicating that SIK2 may lead to MYL2 phosphorylation indirectly.

Previous studies reported that a number of kinases, including ROCKs (ROCK1 and ROCK2), ZIPK, MRCK and MYLK, phosphorylate MYL2 at Ser19 [11, 17]. We determined if these kinases mediate the SIK2‐induced phosphorylation of MYL2. Since ROCK proteins are well‐established upstream kinases of MYL2 phosphorylation [18, 19, 20], we measured their expression levels after SIK2 knockdown. Our results showed no significant change in ROCK1 and ROCK2 proteins (Fig. 2B) indicating that SIK2 does not affect the Rho/ROCK pathway and that SIK2 regulated MYL2 phosphorylation in a ROCK‐independent manner. Co‐immunoprecipitation assay was then employed to determine if SIK2 interacts with these kinases. The results suggested that endogenous SIK2 may interact with endogenous MYLK (Fig. 2C), but not with the other kinases (data not shown). Subsequently, immunofluorescent staining also revealed that SIK2 co‐localized with MYLK (Fig. 2D).

To determine whether SIK2 promoting ovarian cell motility and migration is MYLK dependent, transwell assays were conducted with and without the knockdown of MYLK. The results revealed that MYLK knockdown significantly attenuated cell migration and invasion (Fig. 2E). Furthermore, MYLK silencing reduced the expression of F‐actin and MYL2‐pS19 (Fig. 2F). To determine whether ectopic MYLK expression rescued the effect of SIK2 depletion on the expression of cell motion‐related proteins, OVCAR8 cells with a stable expression of SIK2 shRNA (OVCAR8‐shSIK2) were transfected with control empty vector plasmid (EV) or MYLK overexpressing plasmid (MYLK OE). Results showed that MYLK overexpression abolished the inhibitory effects of SIK2 knockdown on MYL2 phosphorylation and F‐actin expression in OVCAR8 cells (Fig. 2G). Together, these results indicate that SIK2 promotes cell motility and MYL2 phosphorylation through MYLK.

### 3.3 SIK2 phosphorylates MYLK on Ser343

**Fig. 3:**
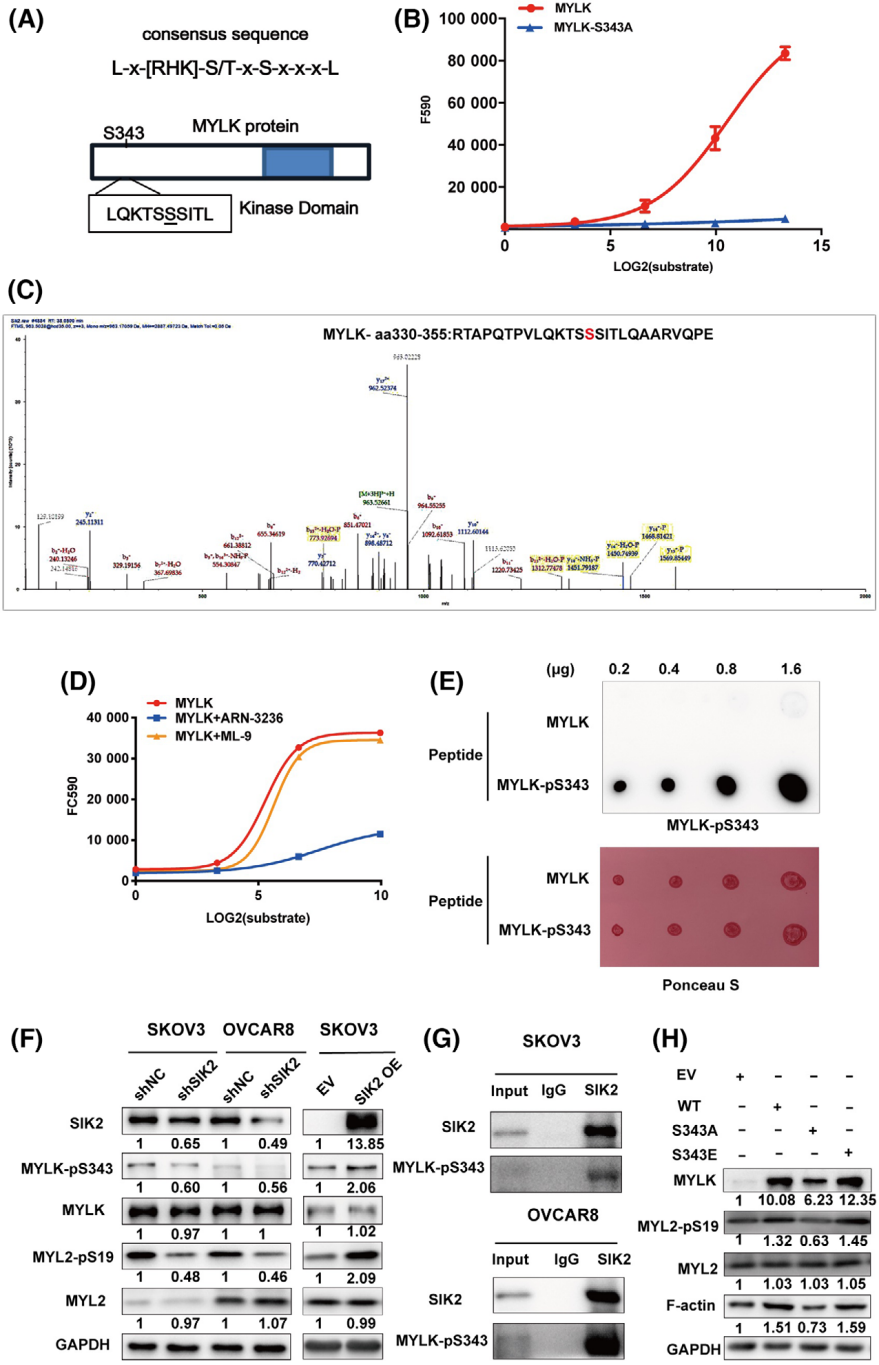
SIK2 phosphorylates MYLK on Ser343. (A) The putative SIK2 phosphorylation site containing the consensus sequence on the MYLK protein. (B) *In vitro* kinase assays were performed using wild‐type or mutant (S343A) MYLK peptide as substrate to identify the phosphorylation site. The error bars indicate SD. (C) LS‐MS/MS assay was performed using the wild‐type or mutant (S343A) MYLK peptide as substrate to identify the phosphorylation site. (D) *In vitro* kinase assay of recombinant SIK2 protein (kinase) and recombinant MYLK protein (substrate) in the presence or absence of the SIK2 inhibitor, ARN‐3236, (1 μm) or the MYLK inhibitor, ML‐9, (2 μm). (E) The synthetic MYLK peptides bearing the S343 residue without phosphorylation (MYLK) or with phosphorylation (MYLK‐pS343) were used in dot blot analysis to determine the validity and specificity of the anti‐MYLK‐p343 antibody. (F) Total and phosphorylated MYLK and MYL2 were detected in cells with SIK2 knockdown or overexpression. (G) Cell lysates were immunoprecipitated with anti‐SIK2 or IgG followed by the detection of MYLK‐pS343 using western blot. (H) Immunoblot analysis of the expression of motion‐related proteins in the OVCAR8‐shSIK2 subclone transfected with MYLK plasmid (WT, S343A, S343E) for 72 h. All the experiments were repeated thrice independently.

To determine the SIK2 phosphorylation site on MYLK, we searched for human proteins containing the putative SIK2 phosphorylation consensus motif LX[HKR]S/TXSXXXL using the SWISS‐PROT database. We identified the peptide sequence LQKTSSSITL corresponding to the amino acids 338‐347 near the N‐terminus of MYLK as a putative SIK2 phosphorylation site (Fig. 3A and Fig. S2A). The *in vitro* kinase assay was performed to determine whether MYLK is a substrate of SIK2 and whether SIK2 phosphorylates MYLK on Ser343. Our results demonstrated that recombinant SIK2 protein directly phosphorylated a 26 amino‐acid peptide (amino acids 330–355) derived from MYLK that contained a putative SIK2 phosphorylation site at the underlined serine amino acid RTAPQTPVLQKTSSSITLQAARVQPE, but not the S343A mutant peptide (Fig. 3B). Furthermore, LC‐MS analysis of the products of *in vitro* kinase assay confirmed that SIK2 led to the phosphorylation of the wild‐type MYLK‐derived peptide but not the S343A mutant peptide (Fig. 3C). In addition, the recombinant SIK2 protein also phosphorylated the recombinant full‐length MYLK protein (Fig 3D). This kinase activity was inhibited by the SIK2 inhibitor (ARN‐3236), but not by the MYLK inhibitor (ML‐9) (Fig. 3D).

To confirm the results of *in vitro* kinase assay and LC‐MS analysis, MYLK‐pS343 specific antibody was generated using the unphosphorylated or S343‐phosphorylated MYLK‐derived peptide (aa 330–355) and validated by dot blot analysis (Fig. 3E). The results indicated that the antibody exhibited high affinity to MYLK‐pS343 peptide, but not to the unphosphorylated peptide. ELISA and western blot analysis also showed the high specificity of the MYLK‐pS343 antibody (Fig. S2B,C). The MYLK‐pS343 antibody was therefore used to determine the effect of SIK2 on MYLK‐pS343 protein expression. The depletion of SIK2 resulted in a significant decrease in MYLK‐pS343 protein expression, while SIK2 overexpression increased MYLK phosphorylation at the Ser343 site in SKOV3 cells (Fig. 3F). Additionally, co‐immunoprecipitation analysis showed that MYLK‐pS343 was immunoprecipitated by anti‐SIK2 antibody but not IgG in SKOV3 and OVCAR8 cell lysates (Fig. 3G), indicating an interaction between MYLK‐pS343 and SIK2. The overexpression of MYLK‐WT and its phosphomimetic mutant MYLK‐S343E abolished the inhibitory effects of SIK2 knockdown on MYL2 phosphorylation and F‐actin expression in OVCAR8 cells with stably expressed SIK2 shRNA. In contrast, the dephosphomimetic mutant MYLK‐S343A was unable to abolish the inhibitory effects of SIK2 knockdown (Fig. 3H). These results suggested that SIK2 directly phosphorylated MYLK on S343.

### 3.4 ARN‐3236 inhibits the MYLK/MYL2 axis and ovarian cancer cell migration and motility

**Fig. 4:**
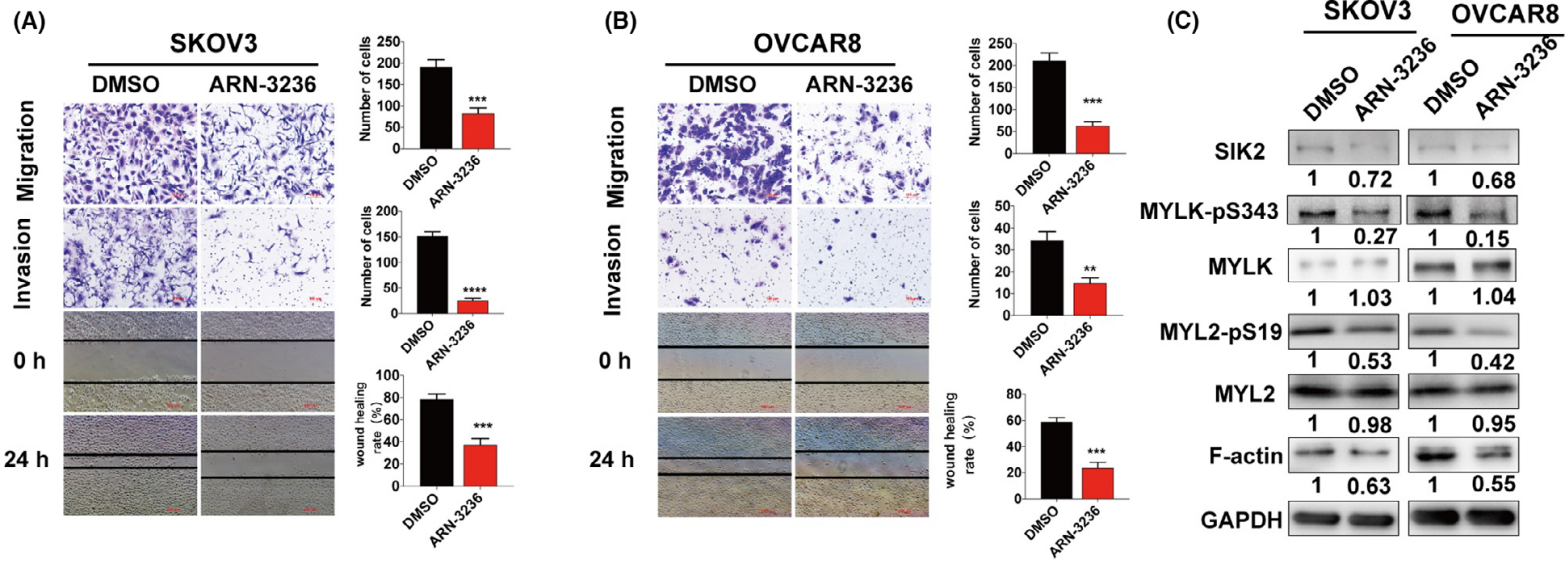
ARN‐3236 inhibits the MYLK/MYL2 axis and ovarian cancer cell motility. (A, B) SKOV3 and OVCAR8 cells were subjected to wound healing and transwell migration and invasion assays in the presence of SIK2 inhibitor, ARN‐3236 (2 μm) or DMSO; the representative images are shown on the left panel, while the quantitative values, which are presented as mean ± SD of three independent experiments are shown on the right panel. Statistical values were calculated by student *t*‐test (unpaired two‐tailed). (C) Immunoblot analysis of F‐actin and MYL2‐pS19 expression in SKOV3 and OVCAR8 after treatment with ARN‐3236 (2 μm) or DMSO for 48 h. All the experiments were repeated in three independent experiments. Bar plots represent the means ± SD (**P < 0.01, ***P < 0.001, ****P < 0.0001).

The SIK2 inhibitor, ARN‐3236, was used to further assess the effect of SIK2 on cancer cell migration and motility. The concentration of ARN‐3236 (2 μm) was chosen such that it had minimal effects on cell proliferation (Fig. S3A,B). ARN‐3236 significantly inhibited the migration and invasion of the ovarian cancer cell lines, SKOV3 and OVCAR8, (Fig. 4A,B) and the breast cancer cell lines, MDA‐MB‐231 and Hs578t (Fig. S3C). Furthermore, ARN‐3236 treatment also led to reduced expression of MYLK‐pS343 and MYL2‐pS19 (Fig. 4C). These results indicated that ARN‐3236 inhibits the MYLK/MYL2 axis and cell motility in ovarian and breast cancer cells.

### 3.5 Omentum‐derived adipocytes promote cell motility and activate the SIK2/MYLK/MYL2 pathway

**Fig. 5:**
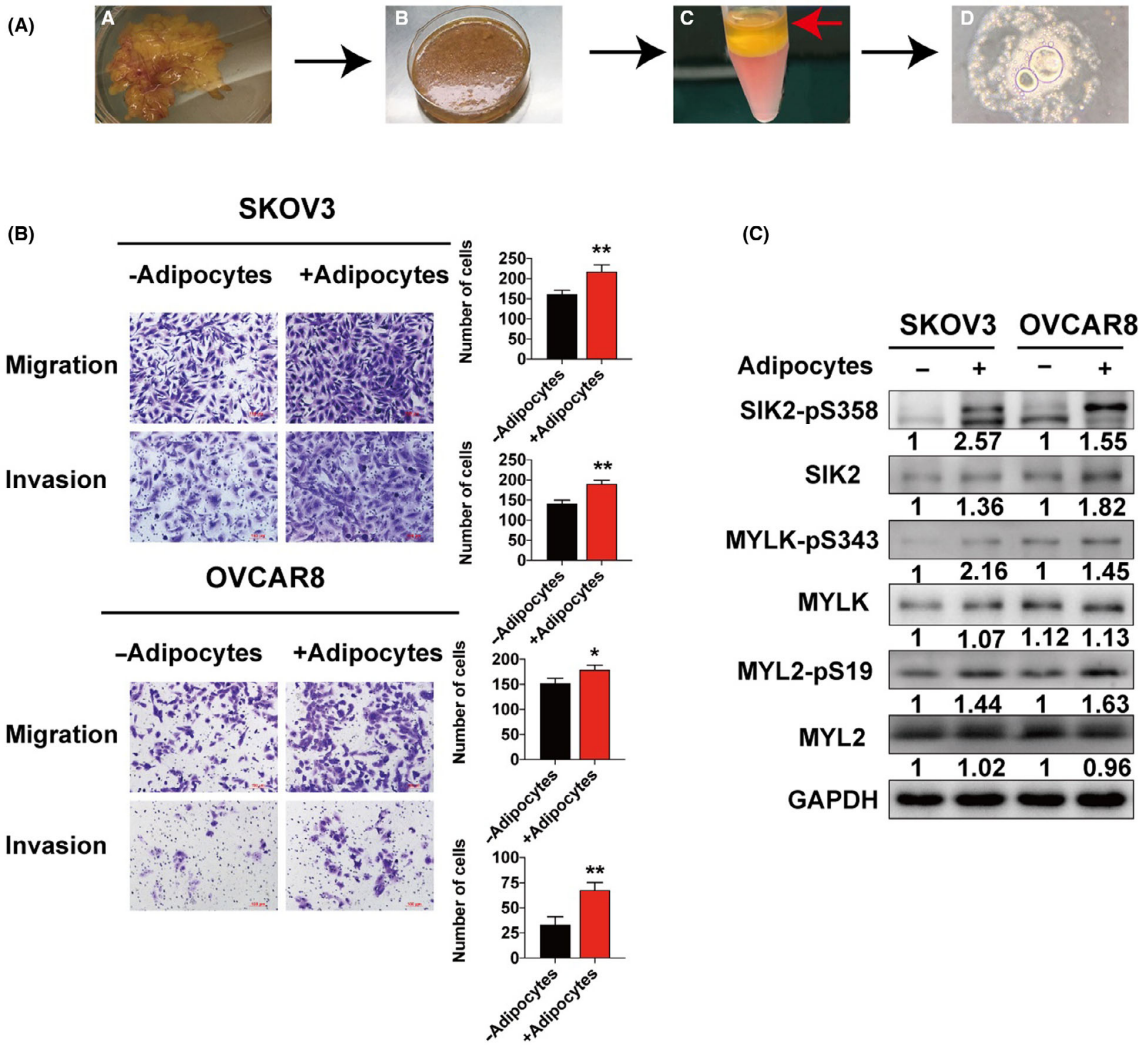
Omentum‐derived adipocytes promote cell motility and activate the SIK2/MYLK/MYL2 pathway. (A) A schematic diagram showing the steps involved in the isolation of adipocytes from the omentum of patients with benign ovarian tumours. (B) SKOV3 and OVCAR8 cells were cultured alone or with adipocytes in the upper chamber, and then transwell assays were performed with or without Matrigel to assess cell migration and invasion capability. The representative images are shown on the left panel, and the quantitative analysis are presented as mean ± SD of three independent experiments. Statistical values were calculated by Student *t*‐test (unpaired two‐tailed). (C) SKOV3 and OVCAR8 cells were cultured alone or with adipocytes for 12 h, and then the cell lysates were used for immunoblot analysis of the indicated proteins. All the experiments were repeated thrice independently. Bar plots represent the means ± SD (*P < 0.05, **P < 0.01).

Previous studies have shown that adipocytes play an important role in promoting ovarian cancer cell migration and invasion [21]. Previous reports indicated that adipocytes regulating cancer metabolism activate SIK2 in ovarian cancer [10]. Hence, we investigated if adipocytes from the omentum increase cancer cell motility and promote cancer cell metastasis. Adipocytes were isolated from metastasis‐free omental tissues collected from patients with early‐stage epithelial ovarian cancer [10] (Fig. 5A). The isolated adipocytes were then co‐cultured with SKOV3 and OVCAR8 cells. Cancer cells co‐cultured with adipocytes showed increased capability of cell migration and invasion in transwell assays, compared to cells cultured alone (Fig. 5B). Cancer cells co‐cultured with adipocytes also showed increased SIK2 phosphorylation at S358, and increased expression of SIK2, MYL2‐pS19 and MYLK‐pS343 (Fig. 5C). These results suggested that the omentum‐derived adipocytes increased cell motility and activated the SIK2/MYLK/MYL2 pathway.

### 3.6 There is a positive correlation between SIK2 and MYLK‐pS343 expression in cancer cells and tissues

**Fig. 6:**
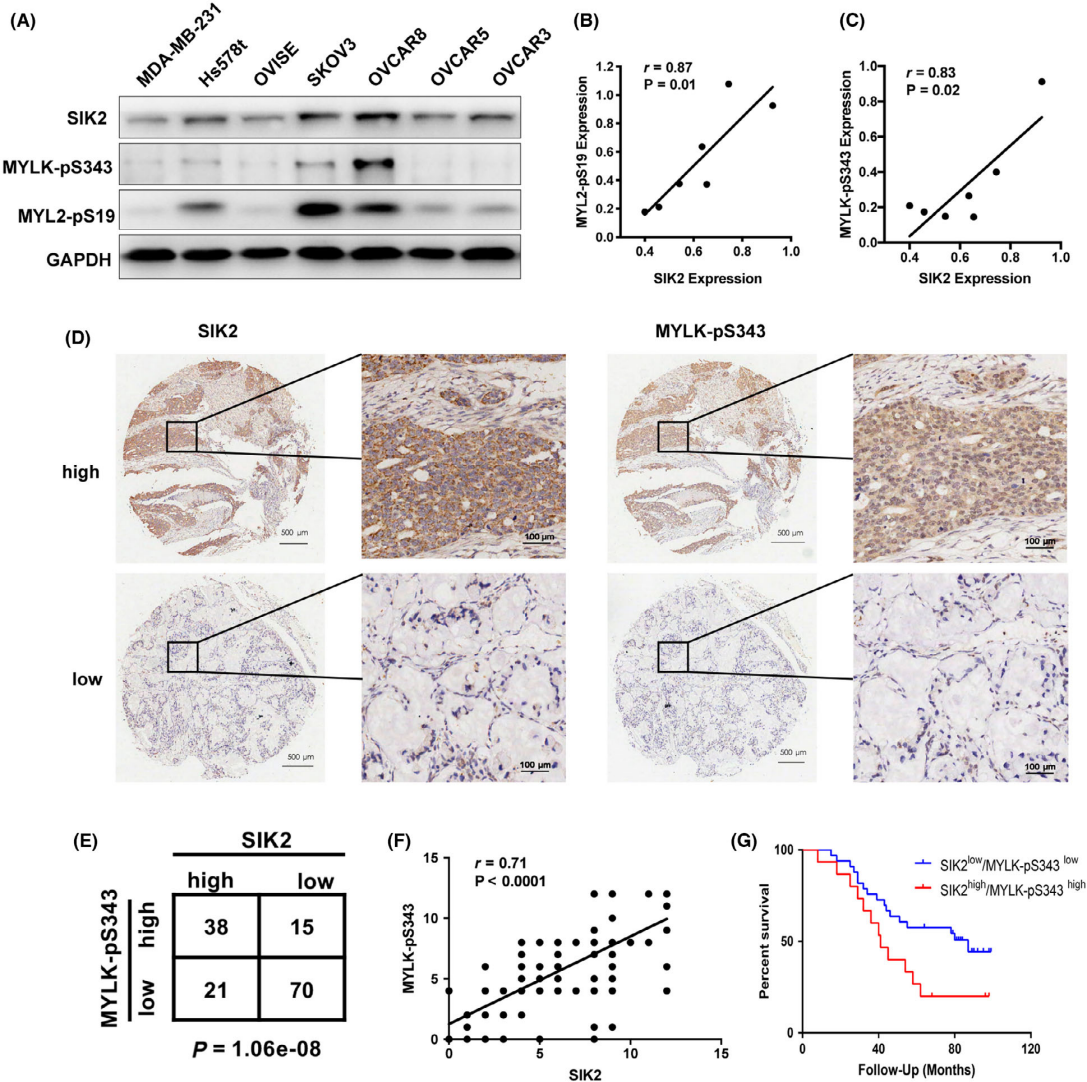
There is a positive correlation between the expression of SIK2 and MYLK‐pS343 in cancer cell lines and tissues. (A) Expression of SIK2, MYL2‐pS19 and MYLK‐pS343 in ovarian cancer cell lines and breast cancer cell lines were detected using western blot. (B) A plot showing the correlation between SIK2 and MYL2‐pS19 in each cell line. Correlations between measured variables were analyzed using Spearman rank correlation test. (C) A plot showing the correlation between SIK2 and MYLK‐pS343 in each cell line. Correlations between measured variables were analyzed using Spearman rank correlation test. (D) Representative images showing immunochemical analysis of paraffin‐embedded, formalin‐fixed ovarian cancer tissue array stained with anti‐SIK2 and anti‐MYLK‐pS343 antibodies; scale bar = 500 μm for 4× and 100 μm for 40×. (E) The correlation between SIK2 and MYLK‐pS343 in ovarian cancer tissue microarray was analyzed using Pearson's chi‐squared test. (F) Quantification and correlation analysis of the expression of SIK2 and MYLK‐pS343 protein in ovarian cancer tissue microarray. Correlations between measured variables were analyzed using Spearman rank correlation test. (G) Kaplan‐Meier curves for overall survival time of FIGO stage III‐IV patients with high co‐expression of SIK2 and MYLK‐pS343 vs low co‐expression of SIK2 and MYLK‐pS343. All the experiments were repeated thrice independently.

We then analyzed the expression levels of SIK2, phosphorylated MYL2 and phosphorylated MYLK in ovarian cancer cell lines (SKOV3, OVCAR8, OVCAR5, OVCAR3 and OVISE) and human breast cancer cell lines (MDA‐MB‐231 and Hs578 t) (Fig. 6A). SIK2 protein expression was positively correlated with the expression of MYL2‐pS19 (*r* = 0.87; Pearson correlation coefficient, *P* = 0.01) (Fig. 6B) and MYLK‐pS343 (*r* = 0.83, Pearson correlation coefficient, *P* = 0.02) (Fig. 6C) in these cancer cell lines.

To evaluate these findings in clinical specimens, a tissue microarray containing 144 epithelial ovarian cancer cases was stained with anti‐SIK2 and anti‐MYLK‐pS343 antibodies for immunohistochemical analysis (Fig. 6D and Table. S1). SIK2 and MYLK‐pS343 were highly expressed in 41% (59/144) and 37% (53/144) of the epithelial ovarian cancer cases, respectively (*P* = 1.06e‐08; Fig. 6E). More importantly, SIK2 expression was also positively correlated with MYLK‐pS343 expression in ovarian cancer tissues (*r* = 0.71, *P* < 0.0001; Fig. 6F), suggesting that tumours with high SIK2 expression were likely to have high MYLK‐pS343 expression. Thereafter, we examined if the expression levels of SIK2 and MYLK‐pS343 were associated with overall survival of FIGO stage III‐IV serous ovarian cancer patients. Kaplan–Meier curves showed that high co‐expression of SIK2 and MYLK‐pS343 was associated with higher ovarian cancer mortality rates (median survival for patients with SIK2^high^/MYLK‐pS343^high^ vs SIK2^low^/MYLK‐pS343^low^ = 41 vs 87 months, hazard ratio [HR] of death from ovarian cancer = 2.44; 95%CI = 1.041 to 5.715, *P* = 0.04) (Fig. 6G). These results indicate that SIK2 expression is associated with MYLK phosphorylation in ovarian cancer.

### 3.7 SIK2 accelerates metastasis of ovarian cancer *in vivo*

**Fig. 7:**
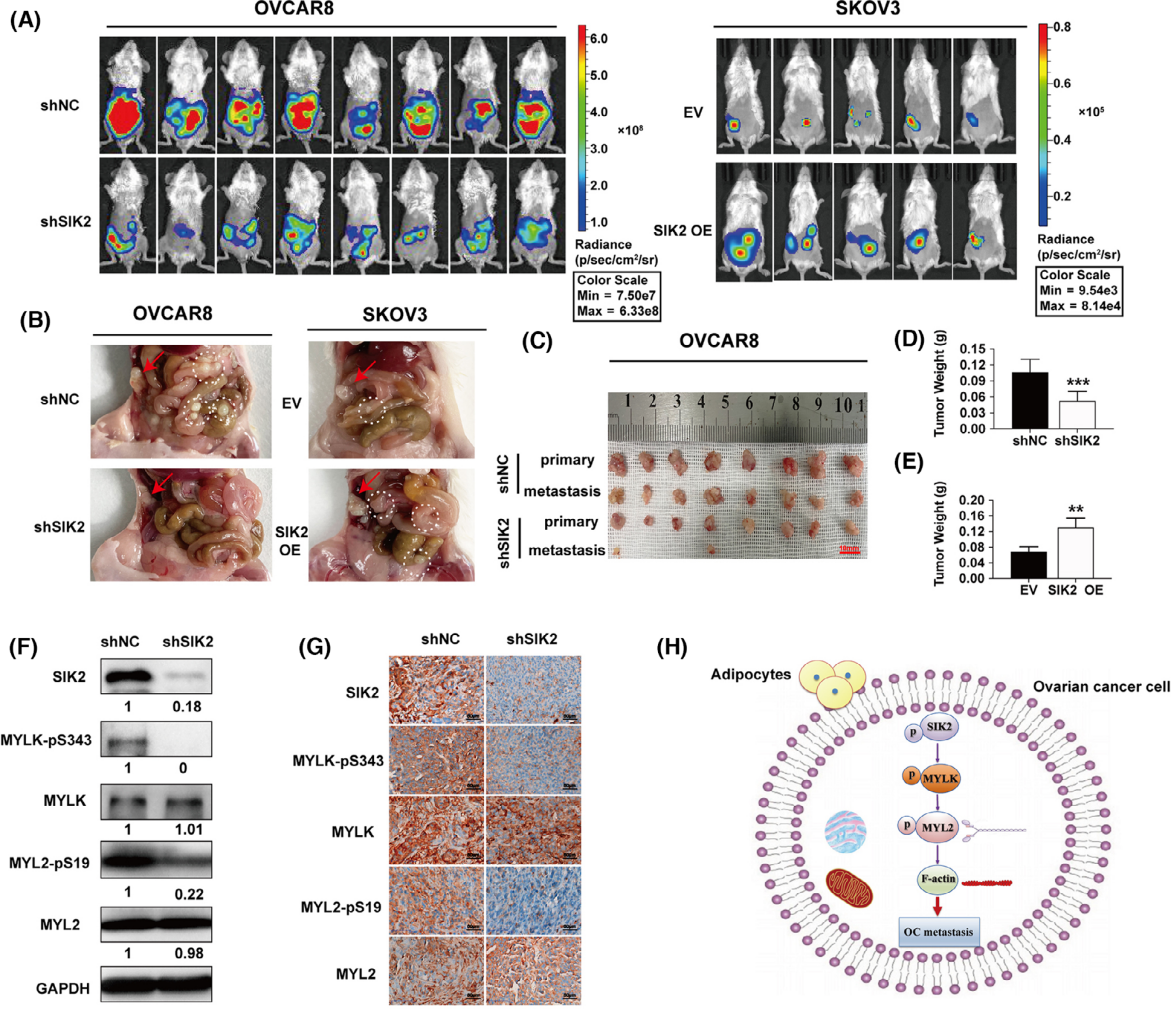
SIK2 accelerates metastasis of ovarian cancer *in vivo* (A) Two pairs of ovarian cancer metastasis orthotopic models (SKOV3‐EV, SKOV3‐SIK2 OE and OVCAR8‐shNC, OVCAR8‐shSIK2 cancer cells) were orthotopically implanted into the right ovarian bursa of C.B‐17 SCID mice. The images show the results of luciferase imaging of the primary and metastatic tumours 4 weeks after implantation. (B) Omental metastasis in mice that were orthotopically transplanted with SKOV3‐EV, SKOV3‐SIK2 OE and OVCAR8‐shNC, OVCAR8‐shSIK2 cancer cells (red arrow represents the primary lesion, whereas the white dotted circle represents metastatic tumours). (C–E) The primary and metastatic tumours of the OVCAR8‐shNC and OVCAR8‐shSIK2 groups. The weight of tumours in each group was also analyzed. Statistical values were calculated by student *t*‐test (unpaired two‐tailed). (F, G) Western blot and immunochemical analysis of the expression levels of SIK2, MYLK, MYLK‐pS343, MYL2 and MYL2‐pS19. (H) A schematic diagram depicting the regulation of cell motility and metastasis by SIK2 in ovarian cancer cells. All the experiments were repeated thrice independently. Bar plots represent the means ± SD. (**P < 0.01, ***P < 0.001).

Two orthotopic models of ovarian cancer metastasis (SKOV3 and OVCAR8 cancer cells) were chosen to evaluate the potential role of SIK2 in cancer cell motility and metastasis *in vivo*. OVCAR8 cells stably expressing non‐targeting shRNA (OVCAR8‐shNC), OVCAR8 cells stably expressing SIK2 shRNA (OVCAR8‐shSIK2), SKOV3 stably expressing empty vector (SKOV3‐EV) and SKOV3 stably overexpressing SIK2 (SKOV3‐SIK2 OE) cell subclones were orthotopically implanted in the right ovary bursa of C.B‐17 SCID mice. Mice implanted with OVCAR8‐shSIK2 exhibited a significantly lower tumour burden and abdominal metastases than mice implanted with OVCAR8‐shNC (Fig 7A,B). In contrast, SKOV3‐SIK2 OE cells formed significantly larger abdominal metastases compared with SKOV3‐EV cells (Fig. 7A,B). These results were consistent with the findings from the analysis of tumour weight (Fig. 7C–E). Next, the expression levels of SIK2, MYLK, MYLK‐pS343, MYL2 and MYL2‐pS19 in tumour lysates were assessed by western blot and immunohistochemistry. The results of the analysis showed that there was a significant decrease in the levels of MYLK‐pS343 and MYL2‐pS19 in tumour lysates from OVCAR8‐shSIK2 cells compared to OVCAR8‐shNC cells (Fig. 7F,G), suggesting that SIK2 regulates MYLK/MYL2 phosphorylation. We also measured these biomarkers on primary masses and metastatic nodules by WB and IHC. We found that the expression of these biomarkers on metastatic nodules were significantly higher than that of primary masses (Fig. S4A,B). Taken together, our data showed that SIK2 accelerates ovarian cancer cell motility and metastasis by promoting MYLK/MYL2 phosphorylation and that targeting SIK2 inhibits ovarian cancer metastasis *in vivo* (Fig. 7H).

## 4 Discussion

We have found for the first time that SIK2 regulates ovarian cancer cell motility and metastasis by directly phosphorylating MYLK at Ser343 and activating MYL2, the downstream effector of MYLK. These findings suggest that SIK2 is a potential therapeutic target for inhibiting ovarian cancer metastasis.

SIK2 is a member of the AMPK family and was first cloned from the adrenal gland of a salt‐treated rat [22]. SIK2 has been shown to play an important role in various biological processes, including gluconeogenesis, neuronal survival, melanogenesis, hepatic steatosis, centrosome splitting, reprogramming of glucose metabolism, as well as fatty acid, cholesterol syntheses and paclitaxel chemosensitivity [23, 24, 25, 26, 27]. Furthermore, SIK2 partially promotes insulin secretion from pancreatic β‐cells after glucose stimulation by phosphorylating p35 [28]. Interestingly, MYLK also enhances glucose‐stimulated insulin secretion through actin remodelling, and MYLK catalytic activity is required for the modulation of insulin secretion by the pancreas [29]. Our study results showed that SIK2 regulates ovarian cancer cell motility and metastasis, and that SIK2 inhibition attenuates metastasis and reduces MYL2 phosphorylation in ovarian cancer.

MYLK regulates MYL2 through phosphorylation at Ser19 [30, 31]. MYL2 phosphorylation promotes cell contraction and motility, leading to changes in the actin cytoskeleton [20]. Rapid and dynamic changes in the cytoskeleton are required for cancer cell invasion and metastasis [15]. Although ROCK has been reported to phosphorylate both Thr18 and Ser19 of MYL2 [32], our findings revealed that SIK2 regulates MYL2 phosphorylation in a ROCK‐independent manner. Previous studies have reported that MYLK regulates cell migration in several cancers, including melanoma, gastric, liver, lung, bladder, prostate and breast cancers [33, 34, 35]. Consistent with these findings, our results indicate that MYLK knockdown diminishes ovarian cancer metastasis, mimicking the effect of SIK2 knockdown.

There are multiple phosphorylation sites that have been identified on MYLK proteins [36]. It has been reported that phosphorylation at Ser1760 and Ser1759 leads to an inhibitory effect on MYLK kinase activity, and c‐Src‐dependent phosphorylation at Tyr464 and Tyr471 increases the enzymatic activity of MYLK [37]. In our study, we identified a novel phosphorylation site (S343) as the direct substrate of SIK2. We found that SIK2 promotes MYLK mediated MYL2 phosphorylation, cell motility and metastasis in ovarian cancer. Our results also revealed that SIK2 directly phosphorylates MYLK at Ser343, suggesting that SIK2 enhances cell motility and metastasis by directly phosphorylating MYLK and activating MYLK/MYL2 signalling in ovarian cancer.

Although our study provides new insights into the role of SIK2 in cell motility and metastasis in ovarian cancer, we did not confirm whether high expression of SIK2 play a similar role in other disease sites. Therefore, the mechanisms by which SIK2 regulates cancer cell motility in other cancer sites need to be further explored.

## 5 Conclusion

Our study showed that SIK2 is highly expressed in ovarian cancer and increases cell motility and metastasis by activating the MYLK/MYL2 pathway. Targeting SIK2 presents a novel therapeutic strategy for preventing ovarian cancer metastasis.

## Conflict of interest

The authors declare no conflict of interest.

## Author contributions

XS, SMB and QTL performed the research, and XS wrote the paper. XJY, JW, XWZ, FQF and LZ analyzed the data. RCB and ZL reviewed the manuscript. LCG, YGC and JHZ designed and conceptualized the study and conformed the authenticity of the raw data. All authors have read and approved the final manuscript.

## Supporting information


**Fig. S1.** SIK2 promotes cell motility and metastasis in ovarian cancer.
**Fig. S2.** SIK2 phosphorylates MYLK on Ser343.
**Fig. S3.** ARN‐3236 attenuates MYLK/MYL2 axis and ovarian cancer cell motility.
**Fig. S4.** SIK2 accelerates tumor metastasis of ovarian cancer *in vivo*.

**Table S1.** The information of the tissue microarray containing 144 epithelial ovarian adenocarcinoma cases.

## Data Availability

The data that supports the findings of this study are available in the article and the supplementary materials.
