# Supplementary material for: SIK2 promotes ovarian cancer cell motility and metastasis by phosphorylating MYLK
Source: Mol Oncol. 2022 Mar 25;16(13):2558–74. doi: 10.1002/1878-0261.13208 (PMC9251837; doi:10.1002/1878-0261.13208)
Supplement: Supplementary file 1 — Fig. S1. SIK2 promotes cell motility and metastasis in ovarian cancer. Fig. S2. SIK2 phosphorylates MYLK on Ser343. Fig. S3. ARN‐3236 attenuates MYLK/MYL2 axis and ovarian cancer cell motility. Fig. S4. SIK2 accelerates tumor metastasis of ovarian cancer in vivo. [file MOL2-16-2558-s002.docx]

**SIK2 promotes ovarian cancer cell motility and metastasis by phosphorylating MYLK**

Xiu Shi^1,2,3#^, Xuejiao Yu^4#^, Juan Wang^1#^, Shimin Bian^1^, Qiutong Li ^1^, Fengqing Fu ^2,3^, Xinwei Zou ^1^, Lin Zhang^1^, Robert C. Bast, Jr.^4^, Zhen Lu^4^, Lingchuan Guo^5*^, Youguo Chen^1,2*^, Jinhua Zhou^1,2*^

1. Department of Obstetrics and Gynecology, The First Afﬁliated Hospital of Soochow University, Suzhou, People’s Republic of China;

2. Clinical Research Center of Obstetrics and Gynecology, Jiangsu Key Laboratory of Clinical Immunology, Soochow University, Suzhou, People’s Republic of China;

3. Jiangsu Institute of Clinical Immunology, The First Afﬁliated Hospital of Soochow University, Suzhou, People’s Republic of China;

4. Department of Imaging Department, The First Afﬁliated Hospital of Soochow University, Suzhou, People’s Republic of China;

5. Department of Experimental Therapeutics, University of Texas M.D. Anderson Cancer Center, Houston, Texas, USA;

6. Department of Pathology, The First Afﬁliated Hospital of Soochow University, Suzhou, People’s Republic of China

#These authors contributed equally.

*Corresponding Authors: Lingchuan Guo ([wangyuhong@suda.edu.cn](mailto:wangyuhong@suda.edu.cn)) , Youguo Chen ([chenyouguo@suda.edu.cn](mailto:chenyouguo@suda.edu.cn)) and Jinhua Zhou ([fyyzjh@suda.edu.cn](mailto:fyyzjh@suda.edu.cn)) .

**Supplementary results**

**
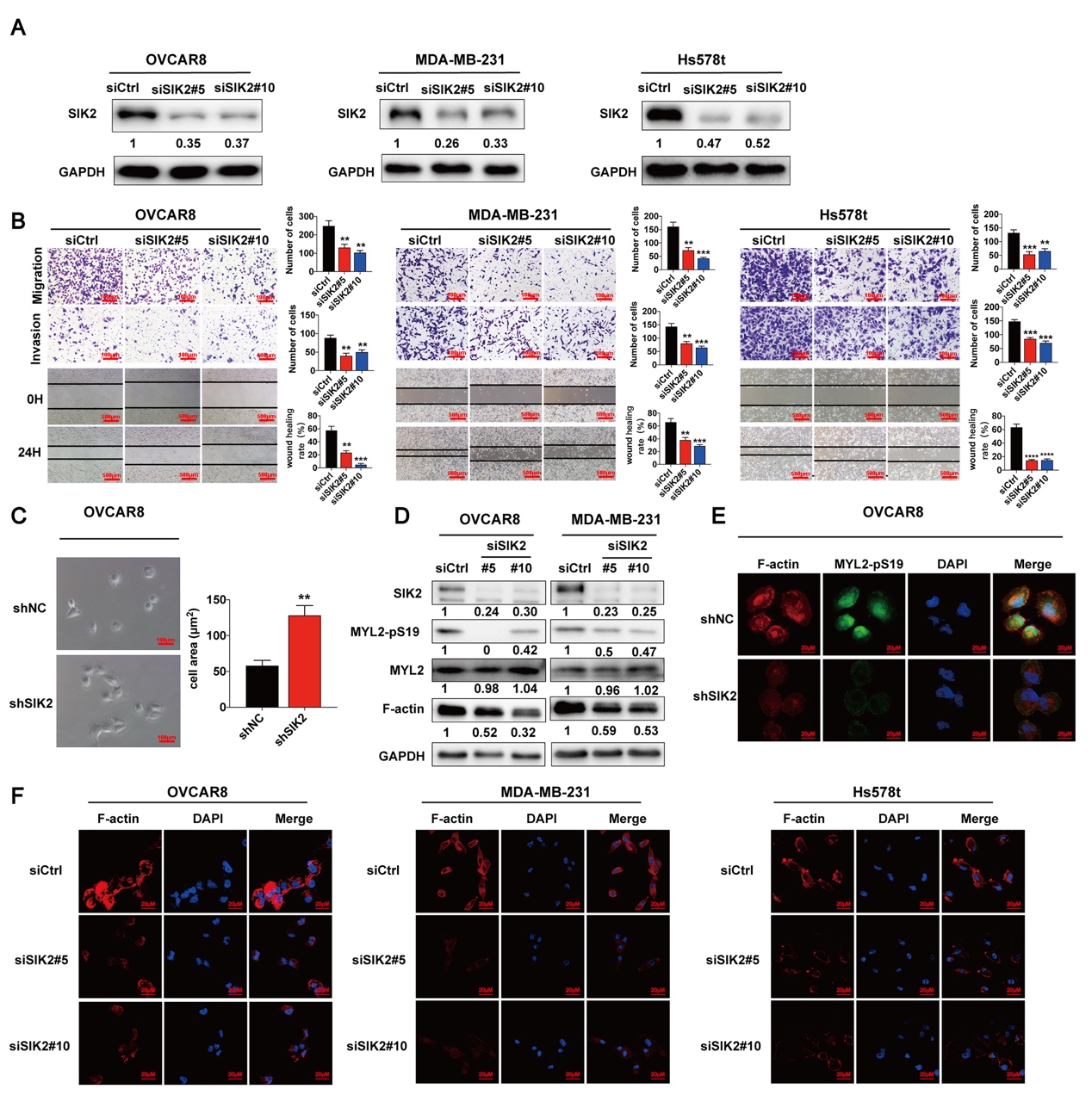
**

**Supplementary Figure S1 related to Figure 1.**

**SIK2 promotes cell motility and metastasis in ovarian cancer.** **A,** Ovarian cancer cell line OVCAR8 and breast cancer cell lines MDA-MB-231 and Hs578t were transfected with SIK2 siRNA for 48 h; knockdown efficiency was identified by Western blotting for SIK2 expression. **B,** After SIK2 siRNA transfection, OVCAR8 cells and MDA-MB-231 and Hs578t cells were subjected to wound healing and transwell migration and invasion assays; the representative images were shown on the left panel, the quantitative values were shown on the right panel as mean ± SD of three independent experiments. **C,** The cell areas of OVCAR8 cell sublcones with stable expression of shSIK2 or shNC were measured by ImageJ, the representative images were shown on the left panel, the areas of 100 cells in each group were measured and shown as means ±SD on the right panel. **D,** Immunoblotting of motion-related proteins F-actin and MYL2-pS19 in OVCAR8 and MDA-MB-231 cells after transfection with SIK2 siRNA for 72 h. **E,** OVCAR8-shSIK2 cells were stained for F-actin (red), MYL2-pS19 (green), and DAPI (blue), OVCAR8-shNC were used as control cells.. **F,** OVCAR8 and MDA-MB-231 and Hs578t cells were transfected with SIK2 siRNA and stained for F-actin (red) and DAPI (blue). (***P*<0.01, ****P*<0.001, *****P*<0.0001). All the experiments were repeated in three independent experiments. Bar plots represent the means ± SD. (**P<0.01, ***P<0.001, ****P<0.0001).


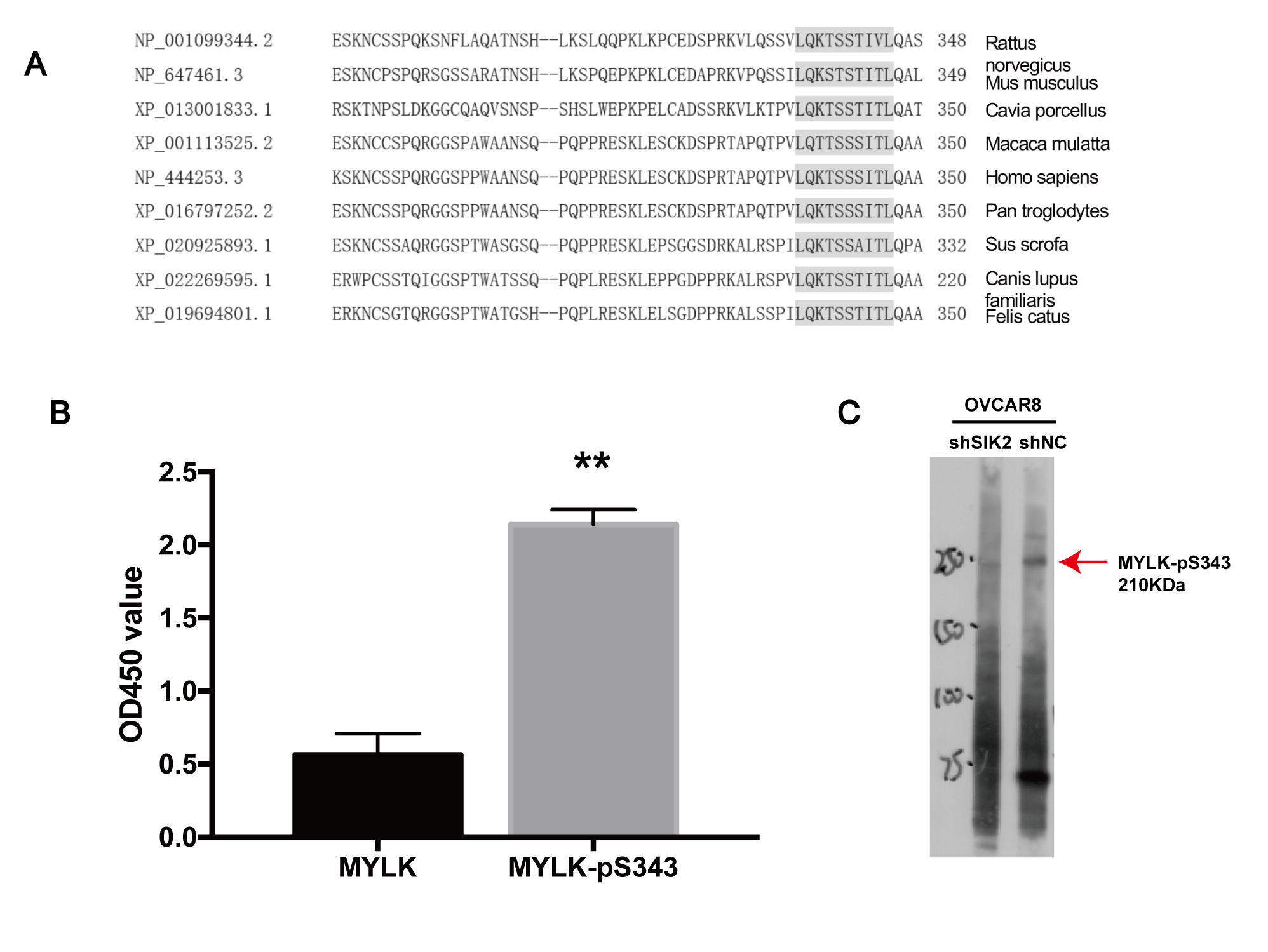


**Supplementary Figure S2 related to Figure 3.**

**SIK2 phosphorylates MYLK on Ser343.** **A,** SIK2 phosphorylated MYLK sequence in different species. **B,** ELISA detected anti MYLK-pS343 antibody interaction with MYLK peptide and MYLK-pS343 peptide. **C,** Detection of MYLK-pS343 antibodies in the protein samples extracted from OVCAR8-shSIK2 and OVCAR8-shNC cells by Western blotting (The red arrow indicates the MYLK-pS343 band). All the experiments were repeated in three independent experiments. Bar plots represent the means ± SD. (**P<0.01).


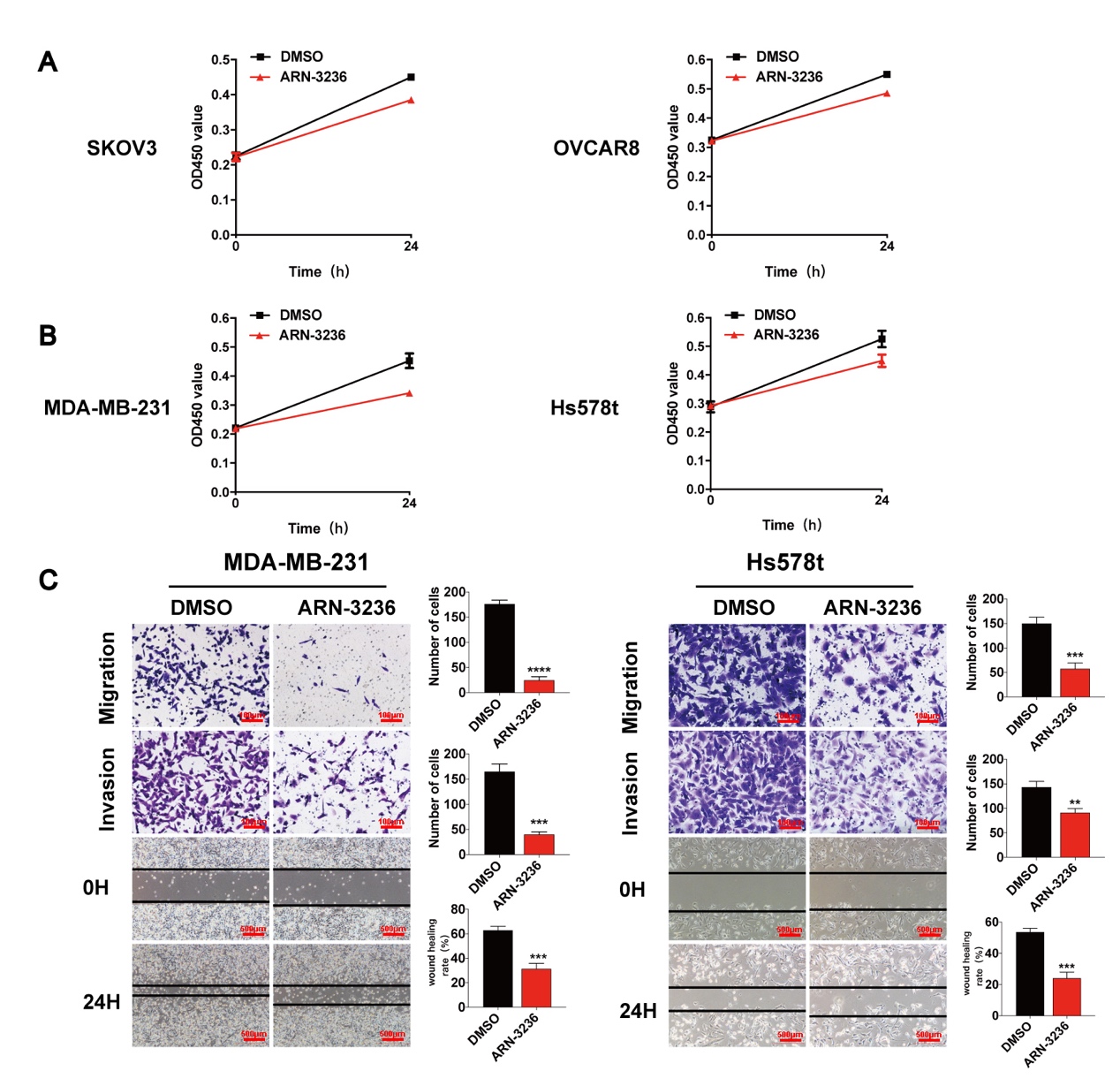


**Supplementary Figure S3 related to Figure 4.**

**ARN-3236 attenuates MYLK/MYL2 axis and ovarian cancer cell motility**

**A and B,** CCK-8 assays were detected in SKOV3 and OVCAR8 and MDA-MB-231 and Hs578t cells after treated with SIK2 inhibitor ARN-3236 (2 μM) for 24h. **C**, MDA-MB-231 and Hs578t cells were subjected to wound healing and transwell migration and invasion assays at the presence of SIK2 inhibitor ARN-3236 (2 μM) or DMSO; the representative images were shown on the left panel, the quantitative values were shown on the right panel as mean ± SD of three independent experiments. All the experiments were repeated in three independent experiments. Bar plots represent the means ± SD. (**P<0.01, ***P<0.001, ****P<0.0001).


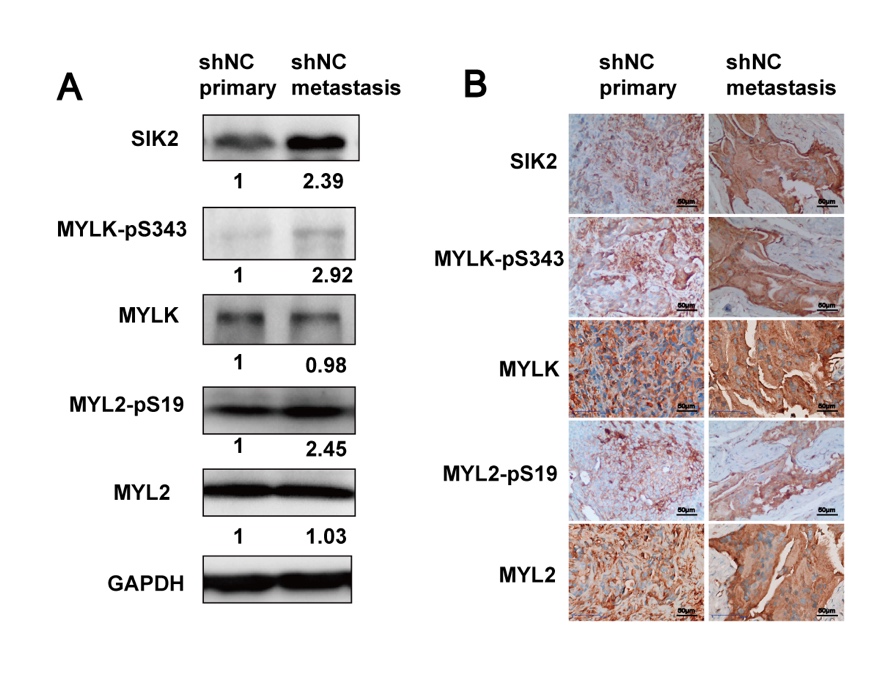


**Supplementary Figure S4** **related to Figure 7**

**SIK2 accelerates tumor metastasis of ovarian cancer *in vivo* A,** The primary and metastatic tumors of the OVCAR8-shNC groups. Western blot analysis of the expression levels of SIK2, MYLK, MYLK-pS343, MYL2, and MYL2-pS19. **B,** The expression levels of SIK2, MYLK, MYLK-pS343, MYL2, and MYL2-pS19 was analyzed by IHC. All the experiments were repeated in three independent experiments.
