# Supplementary material for: SIK2 promotes ovarian cancer cell motility and metastasis by phosphorylating MYLK
Source: Mol Oncol. 2022 Mar 25;16(13):2558–74. doi: 10.1002/1878-0261.13208 (PMC9251837; doi:10.1002/1878-0261.13208)
Supplement: Supplementary file 2 — Table S1. The information of the tissue microarray containing 144 epithelial ovarian adenocarcinoma cases. [file MOL2-16-2558-s001.pdf]

Sample code	Age	Diagnosis	Stage
J01A0867	71	Endometrioid Carcinoma	III
J01A0868	44	Serous Carcinoma	III
D16A6602	47	Serous Carcinoma	IV
J01A0869	46	Serous Carcinoma	III
J01A0870	59	Serous Carcinoma	IV
J01A0871	51	Serous Carcinoma	II
J01A0893	46	Mucinous Carcinoma	III
J01A0872	64	Serous Carcinoma	III
J01A0873	61	Mucinous Carcinoma	IV
J01A0874	36	Serous Carcinoma	II
J01A0875	73	Serous Carcinoma	III
J01A0877	40	Squamous Cell Carcinoma	II
J01A0878	62	Serous Carcinoma	III
J01A0879	51	Serous Carcinoma	III
J01A0880	58	Serous Carcinoma	III
J01A0881	46	Endometrioid Carcinoma	II
D99A0206	61	Serous Carcinoma	IV
J01A0883	45	Mucinous Carcinoma	III
J01A0884	55	Serous Carcinoma	III
J01A0885	62	Serous Carcinoma	IV
J01A0886	40	Serous Carcinoma	III
J01A0887	64	Serous Carcinoma	III
J01A0888	56	Mucinous Carcinoma	I
J01A0890	73	Serous Carcinoma	III
J01A0891	66	Serous Carcinoma	IV
J01A0892	62	Serous Carcinoma	III
J01A0894	58	Endometrioid Carcinoma	I
J01A0895	46	Endometrioid Carcinoma	III
J01A0896	49	Serous Carcinoma	IV
J01A0897	53	Mucinous Carcinoma	III
J01A0898	36	Serous Carcinoma	III
J01A0899	52	Endometrioid Carcinoma	II
J01A0900	56	Serous Carcinoma	IV
J01A0901	20	Mucinous Carcinoma	III
J01A0902	48	Serous Carcinoma	III
J01A0904	47	Serous Carcinoma	III
J01A0905	36	Serous Carcinoma	IV
J01A0906	53	Serous Carcinoma	III
J01A0907	53	Endometrioid Carcinoma	III
J01A0908	49	Serous Carcinoma	IV

J01A0909	52	Serous Carcinoma	III
J01A0910	63	Mucinous Carcinoma	III
D99A0207	73	Serous Carcinoma	IV
J01A0911	65	Mucinous Carcinoma	III
J01A0912	51	Mucinous Carcinoma	III
J01A0913	53	Mucinous Carcinoma	III
J01A0914	57	Serous Carcinoma	III
J01A0915	23	Serous Carcinoma	III
J01A0916	46	Mucinous Carcinoma	IV
J01A0917	25	Serous Carcinoma	IV
J01A0918	56	Endometrioid Carcinoma	III
K04A0062	54	Serous Carcinoma	IV
J01A0919	41	Serous Carcinoma	II
J01A0920	47	Serous Carcinoma	IV
J01A0921	55	Mucinous Carcinoma	II
J01A0923	60	Squamous Cell CarcinomaC	III
J01A0924	56	Mucinous Carcinoma	II
J01A1013	45	Mucinous Carcinoma	I
J01A0926	25	Mucinous Carcinoma	III
J01A0927	37	Mucinous Carcinoma	III
J01A0928	52	Serous Carcinoma	II
J01A0929	56	Serous Carcinoma	IV
J01A0930	73	Mucinous Carcinoma	III
J01A0931	44	Serous Carcinoma	II
J01A0932	60	Clear Cell Carcinoma	III
J01A1014	58	Endometrioid Carcinoma	III
J01A0933	56	Serous Carcinoma	IV
J01A0934	64	Serous Carcinoma	IV
J01A0935	55	Serous Carcinoma	II
J01A0936	37	Mucinous Carcinoma	I
J01A0937	55	Clear Cell Carcinoma	III
J01A0938	64	Serous Carcinoma	III
J01A0939	75	Serous Carcinoma	III
K04A0063	54	Serous Carcinoma	IV
H02A0524	47	Serous Carcinoma	IV
J01A0940	46	Mucinous Carcinoma	IV
A13A0005	69	Serous Carcinoma	IV
J01A0941	46	Clear Cell CarcinomaC	III
J01A0942	48	Serous Carcinoma	III
J01A0943	38	Endometrioid Carcinoma	II
J01A0944	66	Serous Carcinoma	IV
J01A0945	61	Mucinous Carcinoma	III
J01A0946	60	Serous Carcinoma	III

J01A0948	59	Serous Carcinoma	III
A02A0011	55	Serous Carcinoma	IV
J01A0951	41	Serous Carcinoma	IV
J01A0952	37	Mucinous Carcinoma	III
D16A6603	62	Serous Carcinoma	IV
J01A0953	33	Mucinous Carcinoma	III
J01A0954	53	Serous Carcinoma	II
J01A0956	40	Serous Carcinoma	III
J01A0957	39	Endometrioid Carcinoma	II
J01A0958	40	Serous Carcinoma	II
J01A0959	60	Serous Carcinoma	III
J01A0960	39	Serous Carcinoma	II
J01A0961	45	Mucinous Carcinoma	III
J01A0964	44	Serous Carcinoma	III
J01A0965	48	Serous Carcinoma	II
J01A0966	40	Serous Carcinoma	III
J01A0967	43	Serous Carcinoma	II
J01A0968	37	Serous Carcinoma	III
J01A0969	42	Mucinous Carcinoma	II
J01A0970	63	Serous Carcinoma	III
J01A0971	48	Serous Carcinoma	I
J01A0972	55	Mucinous Carcinoma	III
J01A0973	41	Endometrioid Carcinoma	IV
J01A0974	40	Serous Carcinoma	III
J01A0975	25	Mucinous Carcinoma	III
J01A0976	50	Mucinous Carcinoma	II
J01A0977	42	Serous Carcinoma	II
J01A0978	47	Mucinous Carcinoma	I
J01A0979	43	Endometrioid Carcinoma	II
J01A0980	42	Mucinous Carcinoma	II
J01A0981	45	Serous Carcinoma	IV
J01A0982	53	Mucinous Carcinoma	II
J01A0983	43	Serous Carcinoma	III
J01A0984	59	Serous Carcinoma	II
J01A0985	50	Serous Carcinoma	III
J01A0986	57	Serous Carcinoma	IV
J01A0987	46	Serous Carcinoma	III
J01A0988	67	Serous Carcinoma	IV
J01A0990	42	Serous Carcinoma	IV
J01A0991	52	Serous Carcinoma	IV
J01A1015	60	Serous Carcinoma	III
J01A0992	68	Serous Carcinoma	II
J01A0993	56	Serous Carcinoma	III

J01A0994	52	Endometrioid Carcinoma	II
J01A0995	54	Serous Carcinoma	III
J01A0996	66	Serous Carcinoma	III
J01A0997	46	Endometrioid Carcinoma	III
J01A0998	54	Mucinous Carcinoma	II
J01A0999	69	Endometrioid Carcinoma	III
J01A1000	47	Mucinous Carcinoma	III
J01A1016	66	Endometrioid Carcinoma	III
J01A1003	38	Mucinous Carcinoma	III
J01A1004	44	Serous Carcinoma	I
J01A1005	68	Serous Carcinoma	III
J01A1006	66	Serous Carcinoma	II
J01A1007	56	Serous Carcinoma	III
J01A1008	40	Serous Carcinoma	III
J01A1009	42	Serous Carcinoma	I
J01A1010	41	Serous Carcinoma	II
J01A1011	62	Serous Carcinoma	II
J01A1012	38	Endometrioid Carcinoma	III

Supplementary Table 1. **The information of the tissue microarray containing 144 epithelial ovarian adenocarcinoma cases.**
